# Retraction: Ethnomedicinal and folklore inventory of wild plants used by rural communities of valley Samahni, District Bhimber Azad Jammu and Kashmir, Pakistan

**DOI:** 10.1371/journal.pone.0291161

**Published:** 2023-09-05

**Authors:** The PLOS ONE Editors

This article [1] was identified as being linked to a series of submissions for which PLOS noted concerns about authorship and potential competing interests.

In addition, we noted the following concerns:

- The methodology section is missing critical information required for replication studies, including detailed information about the three sites used to collect data. In addition, it is unclear whether the 200 participants interviewed during this study all originated from the same site, or how the results were averaged if these participants originated from different sites.
- Multiple concerns have been raised with the data presented in this study:
  - Fig 3: the methodology section states that 200 informants were interviewed during this study, but the sum of the results reported for each subgroup (ages as well as education level) does not add up to 200.
  - Figs 4, 7, 8, 9, and 10: the size of the error bars presented for each result in these figures appears to be the same and it is unclear what these values represent.
  - Figs 3, 7, 9, and 10: the style of graph used to present the data is an inappropriate selection to relay the type of information presented in these figures.
  - Fig 4 reports on the “percentage of plant parts used in the preparation of TEMs” but the graph includes results that exceed 100%.
  - Fig 6 reports information regarding the “mode of administration of ethnomedicines”, but the results presented include responses such as “powder, paste, decoction, extract, and infusion”, which do not provide any information of the actual mode of administration of these treatments.
  - Fig 7 reports on disease prevalence; it is unclear how the authors determined disease prevalence as the provided questionnaire did not include questions designed to collect data regarding disease prevalence.
  - Multiple figures are of low resolution, and the figure labels do not provide sufficient information to clarify the results presented in the figures.
- Primary data were not provided with the article, contrary to the Data Availability Statement.

The authors provided individual level data underlying the published results. However, editorial assessment of these data raised further concerns regarding inconsistencies in the underlying data, which the authors did not clarify. In addition, the authors did not provide clarifications on how the type of graphs used in the figures were selected or how the error bars presented in this study were calculated, nor did they provide responses to the additional concerns raised with the data presented in this study.

The *PLOS ONE* Editors retract this article due to the unresolved concerns.

MI and TH did not agree with the retraction. MM, MAjaib, MAhmed, IH, HK, WM, SA, KHB, and AG either did not respond directly or could not be reached.
